# Autophagy contributes to apoptosis in A20 and EL4 lymphoma cells treated with fluvastatin

**DOI:** 10.1186/1475-2867-13-111

**Published:** 2013-11-08

**Authors:** Xu-Feng Qi, Dong-Heui Kim, Kyu-Jae Lee, Cheol-Su Kim, Soon-Bong Song, Dong-Qing Cai, Soo-Ki Kim

**Affiliations:** 1 Key Laboratory for Regenerative Medicine of Ministry of Education, Ji Nan University School of Life Science and Technology, Guangzhou, 510632, People’s Republic of China; 2 Department of Developmental & Regenerative Biology, Ji Nan University School of Life Science and Technology, Guangzhou, 510632, People’s Republic of China; 3 Department of Environmental Medical Biology, Yonsei University Wonju College of Medicine, Wonju, Gangwon, 220-701, South Korea; 4 Department of Microbiology, Yonsei University Wonju College of Medicine, Wonju, Gangwon 220-701, South Korea; 5 Institute of Basic Medical Science, Yonsei University Wonju College of Medicine, Wonju, Gangwon 220-701, South Korea

**Keywords:** Fluvastatin, Lymphoma cells, Apoptosis, Autophagy, Mevalonate pathway

## Abstract

Convincing evidence indicates that statins stimulate apoptotic cell death in several types of proliferating tumor cells in a cholesterol-lowering-independent manner. However, the relationship between apoptosis and autophagy in lymphoma cells exposed to statins remains unclear. The objective of this study was to elucidate the potential involvement of autophagy in fluvastatin-induced cell death of lymphoma cells. We found that fluvastatin treatment enhanced the activation of pro-apoptotic members such as caspase-3 and Bax, but suppressed the activation of anti-apoptotic molecule Bcl-2 in lymphoma cells including A20 and EL4 cells. The process was accompanied by increases in numbers of annexin V alone or annexin V/PI double positive cells. Furthermore, both autophagosomes and increases in levels of LC3-II were also observed in fluvastatin-treated lymphoma cells. However, apoptosis in fluvastatin-treated lymphoma cells could be blocked by the addition of 3-methyladenine (3-MA), the specific inhibitor of autophagy. Fluvastatin-induced activation of caspase-3, DNA fragmentation, and activation of LC3-II were blocked by metabolic products of the HMG-CoA reductase reaction, such as mevalonate, farnesyl pyrophosphate (FPP) and geranylgeranyl pyrophosphate (GGPP). These results suggest that autophagy contributes to fluvastatin-induced apoptosis in lymphoma cells, and that these regulating processes require inhibition of metabolic products of the HMG-CoA reductase reaction including mevalonate, FPP and GGPP.

## Introduction

Malignant lymphoma (ML), a heterogeneous disease with highly variable clinical course and prognosis, is the most common type of adult leukemia [1]. Most patients with malignant lymphomas in clinical course are aggressive and soon after diagnosis require intensive treatment [2]. Both the defective balance between pro- and anti-apoptotic molecules and aberrant up-regulation of pro-survival signals had been shown to be related to resistance of malignant lymphoma cells to radiation therapy and chemotherapy [3]. Despite therapies with purine analogs, glucocorticoids, alkylating agents, or monoclonal antibodies appears to be effective, less toxic and more effective drugs are needed to development.

Statins, the inhibitors of 3-hydroxy-3-methyl glutaryl coenzyme A (HMG-CoA) reductase, are used to treat hypercholesterolemia. Convincing evidence from both *in vitro* and *in vivo* data has demonstrated that statins exert pleiotropic actions beyond their lipid-lowering effects, including immune regulation [4] and cancer prevention [5,6]. Statins have been demonstrated to induce cell cycle arrest and cell death in various cancer cells such as multiple myeloma cells [7], pancreatic cancer cells [8], non-small lung cancer cells [9], waldenstrom macroglobulinemia cells [10], and breast cancer MCF-7 cells [11].

Cell deaths include programmed cell death (PCD) and necrosis. Among them, apoptosis is the common form of PCD in multicellular organisms, which appears to be morphologically characterized by cell shrinkage, chromatin condensation, and formation of apoptotic bodies. These processes are influenced by the unbalance of pro- and anti-apoptotic signals regulated by Bcl2-family members [12]. The main biochemical features of apoptosis include caspase activation and DNA fragmentation [12-14]. Apoptosis is induced by various physiological or toxic stimuli such as chemotherapeutic drugs, DNA damage, ultraviolet irradiation, oxidative stress and endoplasmic reticulum stress [13,15].

Except for apoptosis, another cell death model, autophagy, is a bulk degradation system for cytoplasmic components including organelles through the lysosomal pathway, and is characterized by the formation of autophagosomes [16]. Autophagosomes ultimately fuse with lysosomes, thereby generating single-membrane autophagolysosomes and degrading their content. In addition to its basic role in the turnover of proteins and organelles, autophagy has multiple physiological and pathophysiological functions including roles in cell differentiation, immune defense, and cell death [16].

Recent studies have demonstrated that there is a close relationship between autophagy and apoptosis in different cell types or under different pathological conditions. The complex formed by S100 calcium-binding protein A8/A9 can induce apoptosis and autophagy in several cancer cells, such as MCF-7, SHEP and HEK-293. However, S100A8/A9-induced cell death could be partially blocked by the inhibition of autophagy [17]. On the other hand, autophagy can be extensively induced in normal mouse B cells or WEHI-231 B cell line upon induction of BCR ligation-induced apoptosis [18]. These previous reports indicated that the relationship between apoptosis and autophagy varies under different conditions. We previously demonstrated that statins can induce apoptotic cell death in lymphoma cells by stimulating caspase3-related pathways [19]. However, the potential involvement of autophagy in statin-induced apoptosis remains unclear. Here, we reported that fluvastatin induced apoptosis in lymphoma cells by activating pro-apoptotic signals including caspase-3 and Bax and by suppressing anti-apoptotic signal, Bcl2. Furthermore, autophagy was also observed in fluvastatin-treated lymphoma cells. Interestingly, autophagy contributed to fluvastatin-induced apoptosis in lymphoma cells.

## Materials and methods

### Reagents

Fluvastatin (sodium salt, C_24_H_25_FNNaO_4_) was purchased from Calbiochem (La Jolla, CA, USA). Propidium iodide (PI), mevalonate (Mev), farnesyl pyrophosphate ammonium salt (FPP), geranylgeranyl pyrophosphate ammonium salt (GGPP), coenzyme Q10 (CoQ10), and 3-Methyladenine (3-MA) were purchased from Sigma-Aldrich Co. (St. Louis, MO, USA). Antibodies against cleaved caspase-3, Bax, Bcl2, LC3, β-actin and HRP-conjugated goat anti-rabbit IgG were from Cell Signaling Technology (Beverly, MA, USA). A sensitive western blotting luminal reagent was obtained from Santa Cruz Biotechnology Inc. (Santa Cruz, California, USA).

### Cell culture

A20 and EL4 lymphoma cells (Korean Cell Line Bank, Seoul, Korea) were cultured in RPMI 1640 medium (BioWhittaker Inc., Walkersville, MD, USA) containing 10% fetal bovine serum (FBS), 100 U · mL^-1^ penicillin, and 100 μg · mL^-1^ streptomycin (all from BioWhittaker Inc., Walkersville, MD, USA) at 37°C in a 5% CO_2_ incubator. In experiments described below, the medium was exchanged for RPMI 1640 medium containing 2% FBS.

### Annexin V (AV)/propidium iodide (PI) double staining

Phosphatidylserine on the cell surface was detected with Annexin V-FITC Apoptosis Detection Kit (BD Biosciences, NJ, USA) according to the manufacturer’s instructions. In brief, cells were incubated with fluvastatin at concentrations ranging from 0 to 10 μM for 24 h. Collected cells were incubated with FITC-conjugated annexin V and PI for 20 min at room temperature in the dark. The cells were washed with ice-cold PBS, resuspended, and a fraction of the suspension was smeared on a slide. The slide was air dried, mounted with VECTASHIELD® mounting medium, and examined under a DMI 4000 confocal fluorescence microscope (Leica, Wetzlar, Germany). Morphological assessment of apoptosis was performed as follows: bright-green cells (FITC+/PI-), yellow cells (FITC+/PI+), and birght-red cells (FITC-/PI+) were considered to early apoptotic, late apoptotic (secondary necrotic), and necrotic cells, respectively. A total of about 300 cells from four randomly selected fields were counted, and the number of apoptotic cells was expressed as a percentage of the total number of cells scored.

### Transmission electron microscopy (TEM) observation

The transmission electron microscopy (TEM) was utilized for analyzing the ultra-structural images of nucleolus. In brief, treated cells were collected by centrifugation, samples were then fixed in 2.5% glutaraldehyde (Sigma Co., Ltd., USA) for 24 h, washed in 0.1 M phosphate buffer (pH 7.4), post-fixed in 1% osmium tetroxide (Polyscience Co., Ltd., USA) in 0.1 M phosphate buffer (pH 7.4), and subsequently dehydrated in increasing concentrations of alcohol. The samples were impregnated with prophylene oxide (Merck-Schuchardt Inc., Hohenbrunn, Germany) and embedded in epoxy resin embedding media. After a light microscopic examination of semi-thin sections and staining with 2% (w/v) uranyl acetate and 1% (w/v) lead citrate, and then observed with a JEM-1200EX II (Jeol, Japan) transmission electron microscope.

### Western blotting analysis

Collected cells were washed twice with ice-cold PBS and re-suspended in 150 μL RIPA cell lysis buffer (Sigma-Aldrich Co., St. Louis, MO, USA), mixed completely, incubated on ice for 30 min, and followed by centrifugation 12,000 × *g* for 20 min at 4°C. Supernatants were then stored at -80°C until use. Protein concentrations were determined using the Bio-Rad Protein Assay (Bio-Rad Laboratories, Hercules, CA). Equal proteins (30 μg · lane^-1^) were separated on SDS-PAGE and transferred onto polyvinylidene difluoride (PVDF) membrane (Immobilion-P; Millipore, Bedford, MA). Membranes were then blocked with 5% nonfat milk, washed briefly, incubated with primary antibodies at 4°C overnight, and then incubated with corresponding HRP-conjugated secondary antibodies for 1 h at room temperature. Protein bands were visualized by incubating membranes with chemiluminescence reagents before exposure to X-ray film.

### DNA fragmentation assay

After treatment, cells were harvested in a 1.5 mL Eppendorf tube, washed with PBS, and resuspended in 400 μL lysis buffer (10 mM Tris pH 8.0, 10 mM NaCl, 10 mM ethylenediaminetetraacetic acid (EDTA), 1% Sodium Dodecyl Sulfate (SDS), and 100 μg · mL^-1^ Proteinase K) and incubated at 65°C overnight. 75 μL Potassium acetate (8 M) was then added and the samples were incubated at 4°C for 15 min. 750 μL chloroform was added into the Eppendorf tube, which was then mixed vigorously and centrifuged (12,000 × *g*) at room temperature for 10 min. The supernatant was transferred into a new Eppendorf tube and 750 μL ethanol was added, followed by overnight incubation of the sample at -20°C. DNA was acquired by centrifugation (12,000 × *g*, 10 min) of the sample, washed, dried, and dissolved in 50 μL TE buffer (10 mM Tris–HCl, 1 mM EDTA, pH 8.0). Five micrograms of DNA were analyzed on 2.0% agarose gel.

### Statistical analysis

All data are presented as the mean ± SEM of at least three separate experiments. Statistical analysis was performed using one-way ANOVA followed by Dunnett's multiple comparison test. Comparisons between two groups were analyzed using the Student’s *t*-test. A *p* value of less than 0.05 was considered to be statistically significant.

## Results

### Fluvastatin induced apoptosis in lymphoma cells

**Figure 1:**
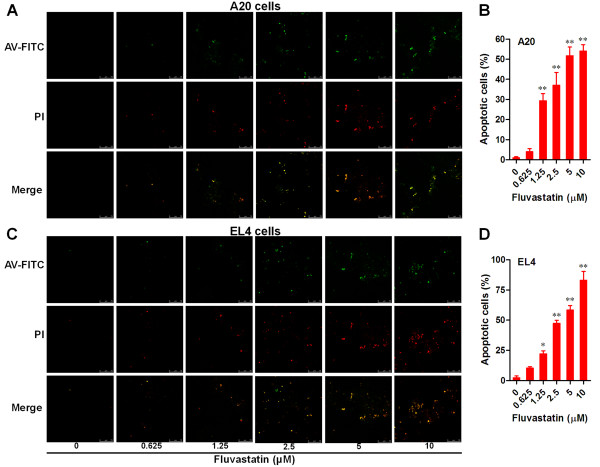
**Fluvastatin induced apoptosis in lymphoma cells.** Lymphoma cells were incubated with fluvastatin (0–10 μM) for 24 h. Apoptosis of lymphoma cells was analyzed by using annexin V (AV)-FITC/PI double staining and confocal laser scanning microscopy. Representative images from A20 cells **(A)** and EL4 cells **(C)** were shown (bar = 75 μm). Apoptosis levels were evaluated by the ratio of apoptotic cells (AV-FITV alone positive and AV-FITC/PI double positive cells) and total cells for A20 cells **(B)** and EL4 cells **(D)**. Data are presented as mean ± SEM of three separate experiments, *p < 0.05, **p < 0.01 versus resting cells.

Apoptosis in lymphoma cells treated with fluvastatin was detected by using annexin V (AV)-FITC/PI double staining and laser confocal fluorescence microscope. Our results showed that the numbers of apoptotic cells (AV-FITC alone positive and AV/PI double positive cells) in both A20 and EL4 cells treated with fluvastatin were greatly increased in a dose-dependent manner (Figure 1A and C). Moreover, the statistic analysis showed that apoptosis of lymphoma cells was significantly induced by fluvastatin even at lowest concentration of 1.25 μM (Figure 1B and D).

### Effects of fluvastatin on activation of apoptosis-related molecules

**Figure 2:**
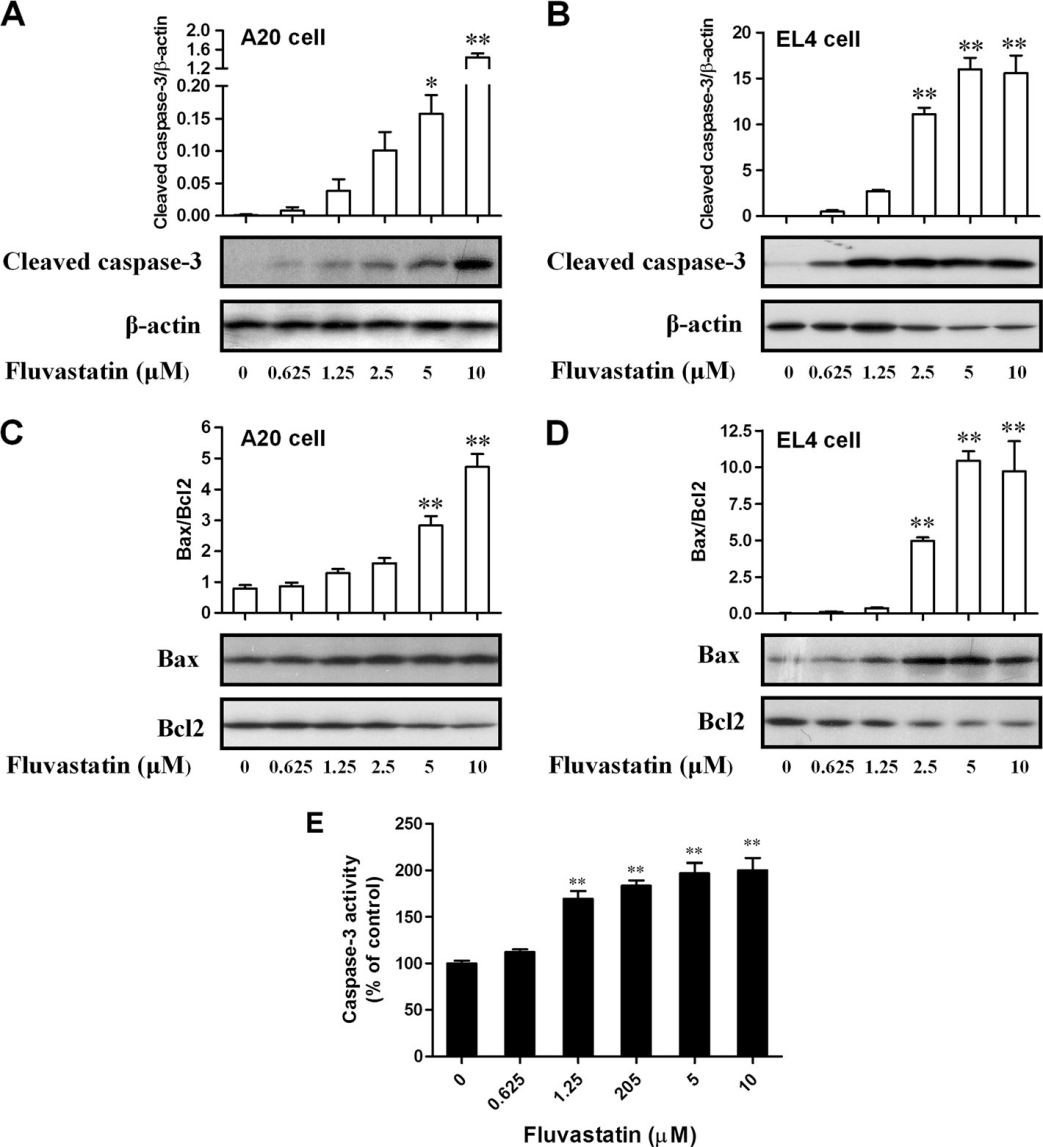
**Effects of fluvastatin on activation of apoptosis-related molecules.** Lymphoma cells were incubated with fluvastatin (0–10 μM) for 24 h. **(A** and **B)** Expression of cleaved caspase-3 in A20 cells **(A)** and EL4 cells **(B)** were examined by using Western blotting. Activation levels of caspase-3 were evaluated by the ratio of chemiluminescent signals between cleaved caspase-3 and β-actin. Representative images were shown in lower panel. Up panel showed the ratio of chemiluminescent signals between cleaved caspase-3 and c Data are presented as mean ± SEM of three separate experiments, *p < 0.05, **p < 0.01 versus resting cells. **(C** and **D)** Expression of Bax and Bcl2 in A20 cells **(C)** and EL4 cells **(D)** were examined by using Western blotting. Apoptosis levels were evaluated by the ratio of chemiluminescent signals between Bax and Bcl2. Representative images were shown in lower panel. Up panel showed the ratio of chemiluminescent signals between Bax and Bcl2. Data are presented as mean ± SEM of three separate experiments, **p < 0.01 versus resting cells. **(E)** Activity of caspase-3 in A20 cells was evaluated by using caspase-3 activity kit. Results are presented as mean ± SEM of three separate experiments, **p < 0.01 versus resting cells.

To further explore the molecular mechanism by which fluvastatin induces apoptosis in lymphoma cells, the activation of several apoptosis relevant molecules was assessed by Western blotting. As shown in Figure 2A and B, fluvastatin increased the expression of cleaved caspase-3 in lymphoma cells in a dose-dependent manner (*p* < 0.05). Moreover, fluvastatin greatly increased the ratio of Bax and Bcl2 in lymphoma cells in a dose-dependent manner (Figure 2C and D). The activity of caspase-3 was also significantly increased in A20 cells exposed to fluvastatin (Figure 2E). These results revealed that the activation of pro-apoptotic molecules including caspase-3 and Bax was increased, but the activation of anti-apoptotic molecule Bcl2 was decreased in fluvastatin-treated lymphoma cells. In addition, increased activity of caspase-3 was also observed in A20 cells treated with fluvastatin.

### Detection of apoptosis and autophagy by TEM in lymphoma cells

**Figure 3:**
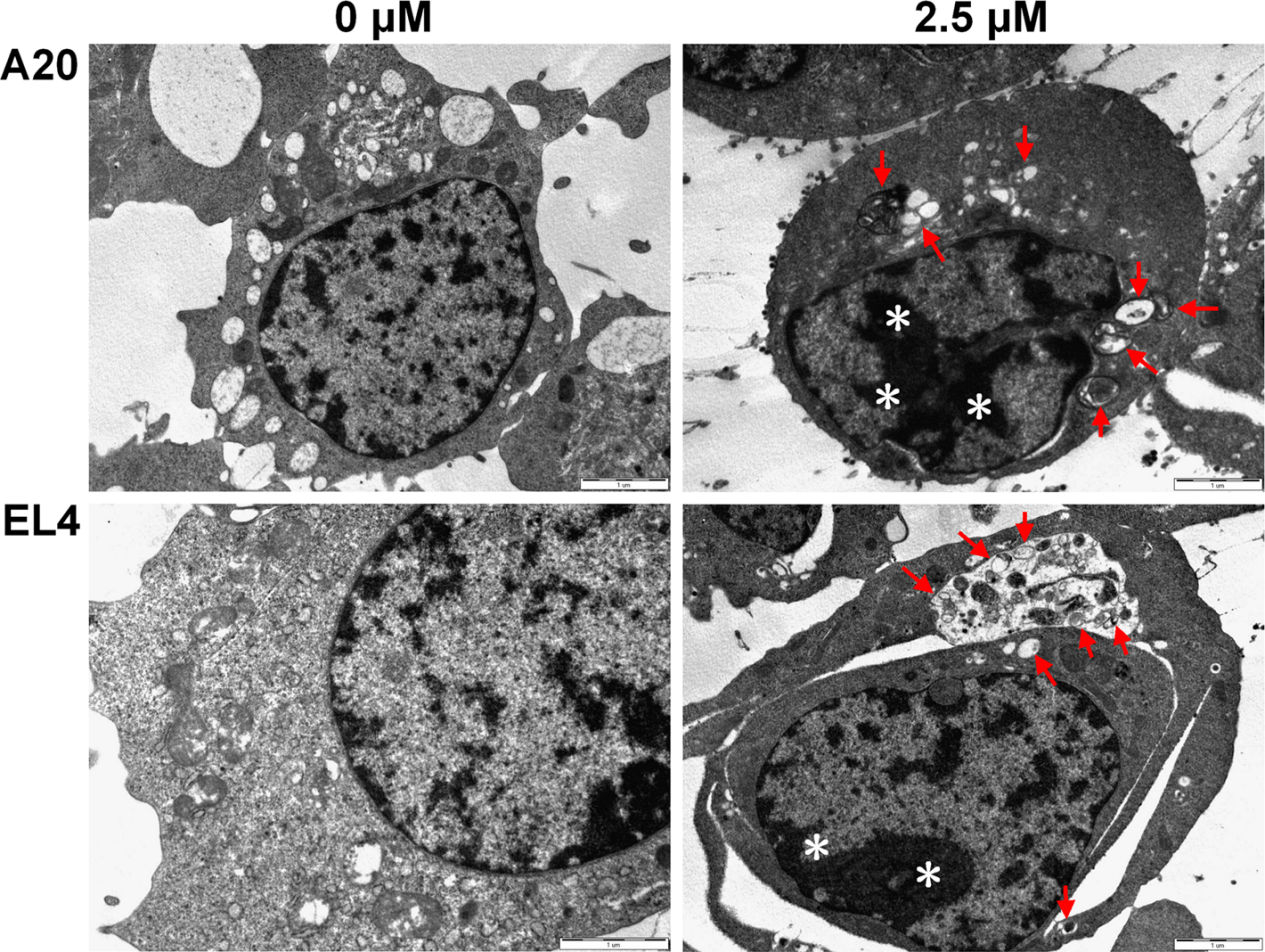
**Detection of apoptosis and autophagy by TEM in lymphoma cells.** Lymphoma cells were incubated with fluvastatin at 2.5 μM for 24 h and analyzed by TEM. Arrows indicate autophagosomes. Asterisks indicate nuclear condensation. Representative images are shown.

Previous study has demonstrated the present of autophagy in apoptotic cells [17,18]. In the present study, to further explore whether autophagy occurs during apoptotic process of lymphoma cells treated by fluvastatin, the transmission electron microscopy (TEM) was utilized for analyzing the ultra-structural images of nucleolus. As shown in Figure 3, autophagosome- or autophagosome-like structures were not observed in unstimulated lymphoma cells. In contrast, lymphoma cells treated with fluvastatin showed autophagosomes and autolysosomes, in which the vacuolar content was degraded (arrows). Furthermore, the features of apoptosis such as chromatin condensation and apoptotic bodies were also observed after treatment with fluvastatin at concentrations of 2.5 μM (asterisks). These findings suggest that autophagy might be involved in apoptosis of lymphoma cells treated with fluvastatin.

### Effects of fluvastatin on activation of autophagy-related molecules

**Figure 4:**
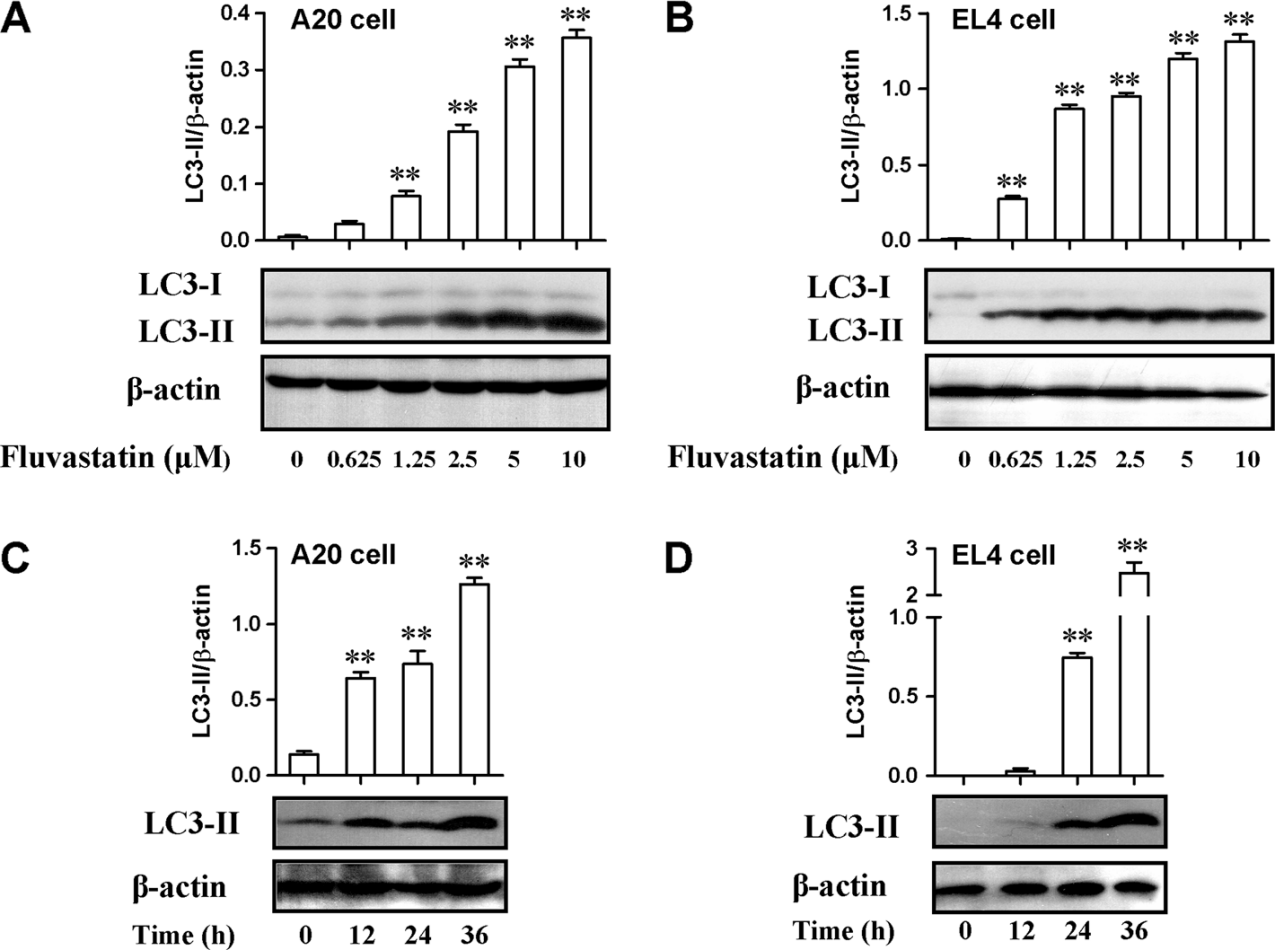
**Effects of fluvastatin on activation of autophagy-related molecules. (A** and **B)** Lymphoma cells were incubated with fluvastatin (0–10 μM) for 24 h. Expression of LC3 in A20 cells **(A)** and EL4 cells **(B)** were examined by using Western blotting. Activation of autophagy was evaluated by the ratio of chemiluminescent signals between LC3-II and β-actin. Representative images were shown in lower panel. Up panel showed the ratio of chemiluminescent signals between LC3-II and β-actin. Data are presented as mean ± SEM of three separate experiments, **p < 0.01 versus resting cells. **(C** and **D)** Lymphoma cells were incubated with fluvastatin (2.5 μM) for indicated periods. Expression of LC3 in A20 cells **(C)** and EL4 cells **(D)** were examined by using Western blotting. Activation of autophagy was evaluated as described above. Representative images were shown in lower panel. Up panel showed the ratio of chemiluminescent signals between LC3-II and β-actin. Data are presented as mean ± SEM of three separate experiments, **p < 0.01 versus resting cells.

The conversion of LC3-I into LC3-II is an essential step in autophagosome formation, and the abundance of LC3-II correlates with the number of autophagosomes [20,21]. To further explore the underlying mechanism for autophagy in fluvastatin-treated lymphoma cells, we examined the expression of LC3-I and LC3-II. As shown in Figure 4A and B, fluvastatin greatly enhanced the expression of LC3-II in A20 or EL4 lymphoma cells 24 h after treatment, in parallel with unchanged or decreased expression of LC3-I, respectively. Moreover, the expression ratio of LC3-II and β-actin in lymphoma cells was significantly increased by fluvastatin in a dose-dependent manner (*p* < 0.01). To explore the time course, we incubated lymphoma cells with fluvastatin at 2.5 μM for 12, 24 and 36 h, respectively. Our results showed that fluvastatin also significantly increased the expression ratio of LC3-II and β-actin in lymphoma cells in a time-dependent manner (Figure 4C and D, *p* < 0.01). These results reveal that fluvastatin could induce autophagy in lymphoma cells by activating LC3-II pathway.

### Fluvastatin induced apoptosis in lymphoma cells through autophagy

**Figure 5:**
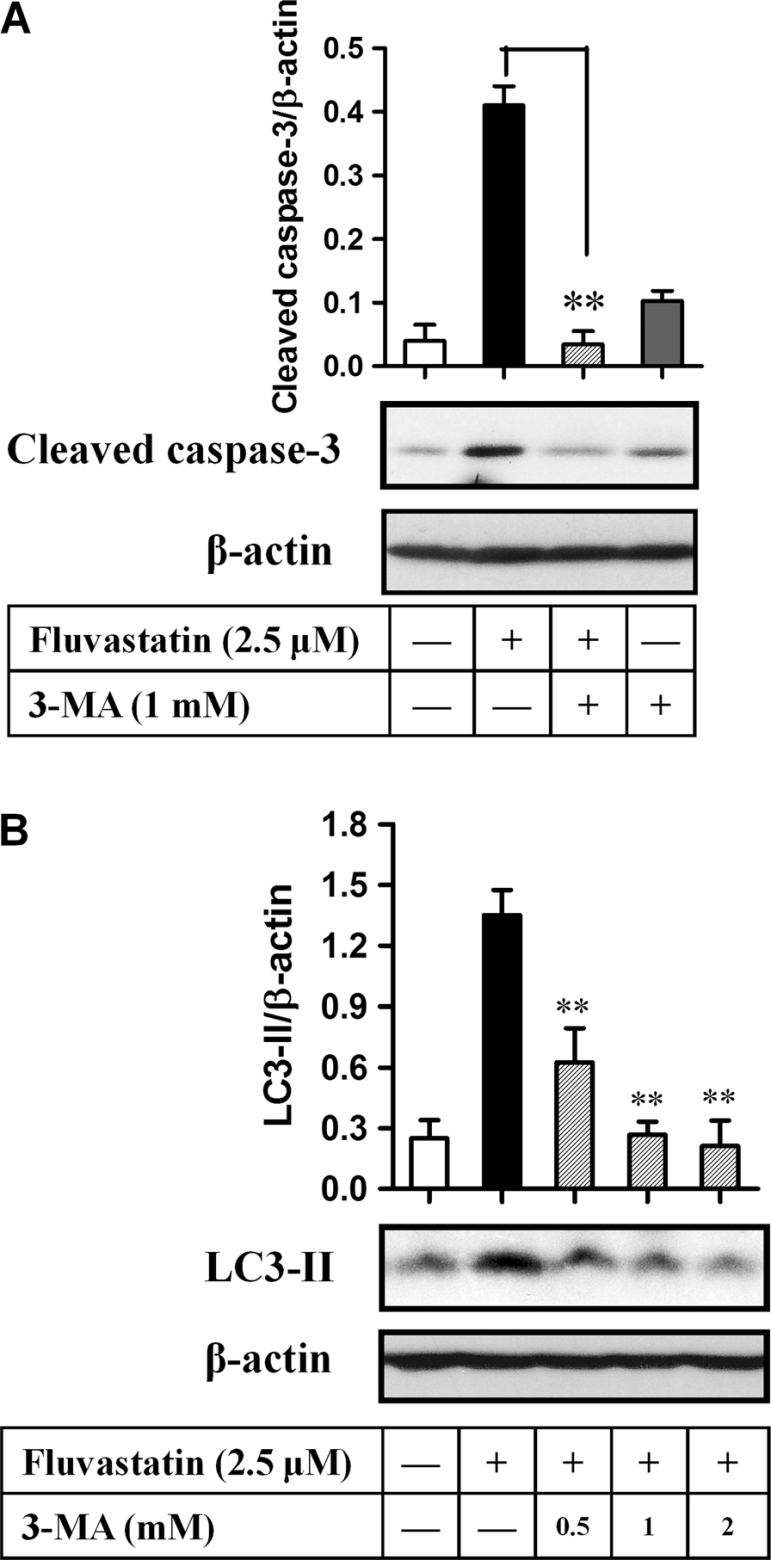
**Fluvastatin induced apoptosis in lymphoma cells through autophagy. (A)** A20 Lymphoma cells were incubated with fluvastatin (2.5 μM) in the presence or absence of 3-methyladenine (3-MA, 1 mM) for 24 h. Expression of cleaved caspase-3 was analyzed by Western blotting. Representative images were shown in lower panel. Activation levels of caspase-3 were evaluated by the ratio of chemiluminescent signals between cleaved caspase-3 and β-actin (up panel). Data are presented as mean ± SEM of three separate experiments, **p < 0.01. **(B)** A20 Lymphoma cells were incubated with fluvastatin (2.5 μM) alone or in the presence of 3-MA (0.5-2 mM) for 24 h, the expression of CL3 was then analyzed. Representative images were shown in lower panel. Activation levels of LC3 were evaluated by the ratio of chemiluminescent signals between LC3-II and β-actin (up panel). Data are presented as mean ± SEM of three separate experiments, **p < 0.01 versus fluvastatin group.

We subsequently examined the relationship between apoptosis and autophagy in lymphoma cells induced by fluvastatin. A20 cells were incubated with fluvastatin at 2.5 μM for 24 h in the presence or absence of the specific inhibitor of autophagy, 3-methyladenine. Activation of caspase-3 was evaluated by the expression ratio of cleaved caspase-3 and β-actin. As shown in Figure 5A, fluvastatin markedly increased the expression of cleaved caspase-3. However, fluvastatin-induced activation of caspase-3 was significantly blocked by the addition of 3-MA (*p* < 0.01), despite no significant changes was observed in lymphoma cells treated with 3-MA alone compared with resting cells. Western blotting analysis also showed that fluvastatin-induced expression of LC3-II was significantly suppressed by the addition of 3-MA in a dose-dependent manner, which confirming the specific inhibition of autophagy by 3-MA (Figure 5B). Taken together, autophagy might contribute to apoptosis in lymphoma cells treated with fluvastatin.

### Fluvastatin-induced apoptosis and autophagy required mevalonate pathways

**Figure 6:**
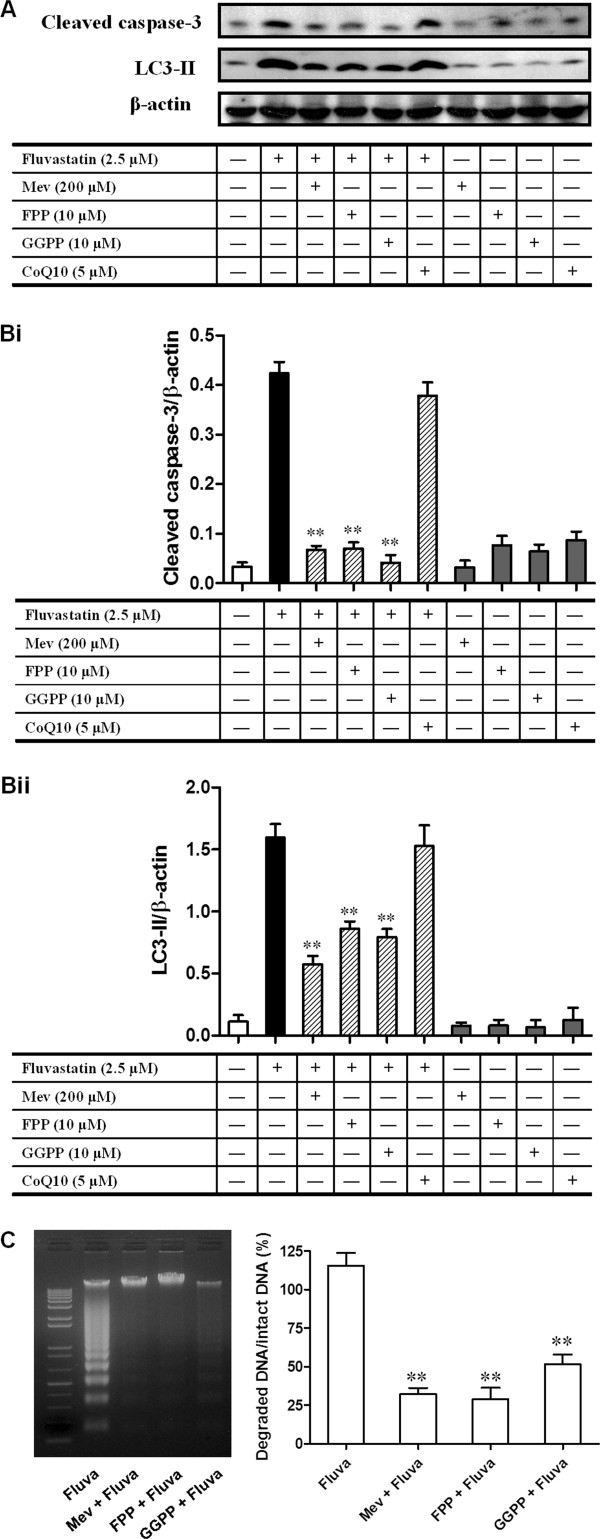
**Fluvastatin-induced apoptosis and autophagy were blocked by mevalonate, FPP, and GGPP. (A and B)** A20 lymphoma cells were incubated with fluvastatin (2.5 μM) in the presence or absence of mevalonate (Mev), farnesyl pyrophosphate (FPP), geranylgeranyl pyrophosphate (GGPP), and coenzyme Q10 (CoQ10) for 24 h. The expression of cleaved caspase-3 and LC3 were then detected by Western blotting. **(A)** Representative images were shown. **(B)** Activation of cleaved caspase-3 **(Bi)** and LC3 **(Bii)** were evaluated as described above. Data are presented as mean ± SEM of three separate experiments, **p < 0.01 versus fluvastatin group. **(C)** A20 lymphoma cells were incubated with fluvastatin (2.5 μM) in the presence or absence of Mev (200 μM), FPP (10 μM), and GGPP (10 μM) for 24 h. DNA fragmentation was then analyzed as described in the Methods section. Left panel showed the representative image of DNA fragmentation. The DNA fragmentation levels were evaluated by the signal intensity ratio between degraded DNA bands versus intact DNA bands (right panel). Data are presented as mean ± SEM of three separate experiments, **p < 0.01 versus fluvastatin group.

To further examine the signaling mechanism for fluvastatin-induced apoptosis and autophagy in lymphoma cells, we incubated A20 cells with fluvastatin in the presence or absence of mevalonate (Mev), geranylgeranyl pyrophosphate ammonium salt (GGPP), farnesyl pyrophosphate ammonium salt (FPP), or coenzyme Q10 (CoQ10). As shown in Figure 6A and B, the increases in expression of cleaved caspase 3 and LC3-II regulated by fluvastatin were markedly blocked by Mev, FPP, or GGPP, but not by the addition of CoQ10. Furthermore, fluvastatin-induced DNA fragmentation in A20 cells was also suppressed by the addition of Mev, FPP, or GGPP (Figure 6C). These results indicated that mevalonate pathways may contribute to fluvastatin-mediated apoptosis and autophagy in lymphoma cells.

## Discussion

Increasing evidence from both *in vitro* and *in vivo* data indicated that statins exhibit cytotoxicicy towards cancer cells by inducing apoptosis in a cell type-dependent manner. Recent studies have demonstrated the complex relationship between autophagy and apoptosis in different cell types [17,18]. However, the involvement of autophagy in fluvastatin-induced apoptosis of malignant lymphoma cells and the potential mechanism are still not clear. In the present study, we revealed that fluvastatin induced 3apoptosis in lymphoma cells by regulating autophagy through inhibition of metabolic products of the HMG-CoA reductase reaction including mevalonate, farnesyl pyrophosphate (FPP) and geranylgeranyl pyrophosphate (GGPP).

Many studies have reported that statins can induce apoptosis in various cancer cells in a cell type-dependent manner [7,9,11]. These reports are consistent with our results showing that fluvastatin induced significant apoptosis in lymphoma cells in a dose-dependent manner, which were examined by annexin V and PI double staining. Increasing knowledge about apoptotic processes has identified several targets which can be used as specific cell death markers, including the changes in mitochondrial membrane potential, cytochrome C, caspase members and so on. The combination of released cytochrome C and apoptotic protease activating factor-1 will active caspase cascade and apoptosis [22,23]. Despite we did not directly examined the release of cytochrome C, our results demonstrated a dose-dependent increase in the numbers of annexin V positive or annexin V/PI double positive cells and the activation of caspase-3 in fluvastatin-treated lymphoma cells (Figures 1 and 2). These results indicating that fluvastatin in deed induces apoptotic death of lymphoma cells. It is well known that the apoptosis is mainly determined by a defective balance among pro- and anti-apoptotic members of the Bcl-2 family, often related to resistance of lymphoma cells to chemotherapy [24]. In our experimental condition, the expression ratio of Bax and Bcl2 was significantly increased in fluvastatin-treated lymphoma cells, indicating that the pro-apoptotic signals were enhanced and the anti-apoptotic signals were suppressed.

Autophagy induced by apoptotic stimuli was mostly found in cancer cells or under the condition where apoptosis is present [17,18]. In the present study, we found that both apoptosis and autophagy were induced by fluvastatin in lymphoma cells. TEM analysis confirmed the presence of ultra-structural characteristics of apoptosis and autophagy in lymphoma cells treated with fluvastatin (Figure 6). The formation of autophagosome requires Atg12 and Atg8 systems, which are tightly associated with the expansion of autophagosomal membrane. Microtubule-associated protein 1-light chain 3 (LC3) is the mammalian homolog of the yeast protein Atg8. Upon synthesis, LC3 is processed to its cytosolic form LC3-I that is subsequently conjugated to the lipid phosphatidylethanolamine, generating the LC3-II form. Conjugation to this lipid is required for its association with the autophagosomal membrane. Which were consistent with our data showing that the levels of LC3-II were significantly increased in lymphoma cells treated with fluvastatin in a dose- and/or time-dependent manner (Figure 4). Taken together, both apoptosis and autophagy can be induced in fluvastatin-treated lymphoma cells. According to previous studies, the relationship between autophagy and apoptosis is cell type- or condition-dependent. On the one hand, autophagy is require for apoptosis in certain cell types with indicated condition. For instance, inhibition of autophagy abrogates tumor necrosis factor alpha induced apoptosis in human T-lymphoblastic leukaemic cells [25]. On the other hand, inhibition of apoptotic pathways cam induce autophagy. For example, combination with inhibition of caspase-3, inhibition of tumor necrosis factor-related apoptosis-inducing ligand (TRAIL) resulted in autophagy [26]. However, our results revealed that fluvastatin-induced activation of cleaved caspase-3 in lymphoma cells was significantly blocked by the addition of specific inhibitor of autophagy, 3-methyladenine (3-MA) (Figure 4), indicating the requirement of autophagy for apoptosis induced by fluvastatin.

Inhibition of HMG-CoA reductase by statins can lower cholesterol by blocking the mevalonate pathway. Besides reducing cholesterol biosynthesis, inhibition of mevalonate by statins also leads to a reduction in the synthesis of isoprenoids such as FPP and GGPP [27]. However, these intermediates are involved in the positive modulation of several non-steroid isoprenoids that are related to antioxidant status, and a reduction in these non-steroid isoprenoids induces oxidative stress [28,29]. Coenzyme Q10 (CoQ10), an important intracellular antioxidant, has membrane stabilizing effects and plays an important role in cellular respiration and defending proteins from oxidation [30]. In addition, dolichol is a polyprenol compound that is synthesized by the general isoprenoid pathway from acetate via mevalonate and farnesyl pyrophosphate and is broadly distributed in membranes. Dolichol functions as a free-radical scavenger in the cell membranes, and may interact with Vitamin E and poly unsaturated fatty acids (PUFA) to form a highly matched free-radical-transfer chain whose malfunctioning might be involved in statin toxicity [31]. In view of these previous studies, it is hypothesized that treatment with statin increases intracellular oxidative stress by disrupting the antioxidant defense system in certain transformed and cancer cells, particularly by inhibiting biosynthesis of isoprenoid antioxidants such as CoQ10 and dolichol. This idea is further supported in part by our results showing that both apoptosis and autophagy induced by fluvastatin were greatly reversed by the addition of Mev, FPP, and GGPP, although CoQ10 had minimal effect on fluvastatin-induced apoptosis and autophagy (Figure 6).

In conclusion, the present study demonstrates that autophagy contributes to fluvastatin-induced apoptosis in malignant lymphoma cells. Furthermore, these regulating processes require the inhibition of metabolic products of the HMG-CoA reductase reaction including mevalonate, farnesyl pyrophosphate and geranylgeranyl pyrophosphate. This study may provide new insight into molecular mechanism by which fluvastatin induces apoptosis of lymphoma cells.

## Competing interest

There are no declared competing interests relevant to this study.

## Authors’ contributions

Conceived and designed the experiments: XFQ, KJL and SKK. Performed the experiments: XFQ. DHK, CSK, SBS and DQC. Wrote the paper: XFQ and SKK. All authors read and approved the final manuscript.
